# The Effects of 17 Weeks of Ballet Training on the Autonomic Modulation, Hormonal and General Biochemical Profile of Female Adolescents

**DOI:** 10.1515/hukin-2015-0062

**Published:** 2015-10-14

**Authors:** Carla Cristiane da Silva, Tamara Beres Lederer Goldberg, Lúcio Flávio Soares-Caldeira, Ricardo dos Santos Oliveira, Solange de Paula Ramos, Fábio Yuzo Nakamura

**Affiliations:** 1 Departamento de Educação Física, Universidade Estadual do Norte do Paraná- (UENP), Jacarezinho, Paraná, Brasil.; 2 Departmento de Pediatria, Curso de Medicina do Adolescente, Programa de Pós-Graduação em Ginecologia, Obstetrícia e Mastologia, Faculdade de Medicina de Botucatu, Universidade do Estado de São Paulo (UNESP), São Paulo, Brasil.; 3 Grupo de Estudos das Adaptações Fisiológicas ao Treinamento- GEAFIT, Londrina, Brasil.; 4 Departamento de Histologia, Universidade Estadual de Londrina, Brasil.

**Keywords:** body composition, autonomic nervous system, ballet training, youth dancers

## Abstract

This study aimed to examine the alterations in physiological and biochemical markers, after 17 weeks of ballet training in high level ballet dancers. Twenty four female ballet dancers from 12 to 15 years old took part in the study. The study followed 17 weeks of ballet training and analyzed changes in body composition, the autonomic nervous system and biochemical variables before and after (post) training. The internal training load was obtained using the session rating of perceived exertion (session-RPE) method, calculated as the mean weekly session-RPE, monotony and strain. After 17 weeks of training there were significant increases in body mass, height, lean body mass, total protein, urea, hemoglobin concentration, testosterone and thyroxine. During this period, decreases in relative body fat, uric acid, red blood cells, C-reactive protein, and ferritin were also found. After the training period, the autonomic modulation demonstrated significant positive alterations, such as increases in parasympathetic related indices. Based on the results obtained we concluded that ballet training led to improvements in body composition and autonomic modulation. In general hematological and biochemical variables demonstrated that the training did not have adverse effects on the health state of the adolescents.

## Introduction

A high performance level in classical ballet depends on a large number of complex components such as technical, artistic and aesthetic elements, associated with highly developed physical and psychological skills (Kuno et al., 1996). This type of dance is characterized by rigid technical movements such as pivots, jumps and poise, along with high levels of flexibility (Kuno et al., 1996). Furthermore, ballerinas must demonstrate grace, self-control and pacing to music (Guidetti et al., 2007; Bria et al., 2011). During training, ballet dancers undertake training sessions composed of intermittent exercises of different durations, interspersed by a wide range of rest periods. Additionally, ballet exercises include coordinated sequences of both static and dynamic whole body muscle actions (Kuno et al., 1996; Roemmich et al., 2001; Guidetti et al., 2007).

In order to achieve high levels of performance, ballerinas engage in about 8 years of full-time training (Guidetti et al., 2007), beginning during childhood and extending throughout adolescence (11–18 years) (Eliakim et al., 2000; Steinberg et al., 2006; Guidetti et al., 2007; Guidetti et al., 2008). The elevated training volume (~10 hours per week), which imposes high demands on the aerobic and anaerobic systems (Guidetti et al., 2007), coupled with a restrictive diet in order to maintain low levels of body fat, are typical exigencies of this discipline (Kuno et al., 1996). However, none of the aforementioned studies monitored the ballerinas’ internal training loads. Therefore, the internal training loads of ballerinas during periods of elevated training volume remain to be determined.

Studies on other modalities have shown that regular training routines lead to positive adaptations of the immune, endocrine (Nemet et al., 2012) and autonomic nervous systems (i.e., an improved vagal modulation (Vinet et al., 2005)). In spite of some inconsistencies in the literature (Eliakim et al., 2013), there is evidence to suggest that the autonomic control of the heart is sensitive to training loads and subsequent adaptations in children and adolescents (Triposkiadis et al., 2002; Vinet et al., 2005). For instance, Sartor et al. (2013) found positive effects of training on heart rate variability in young elite male gymnasts. On the other hand, a bell-shaped relationship between training loads and HRV was observed by Manzi et al. (2009), who showed that increases in training loads beyond the optimal loading range in endurance athletes reflected in a decrease in heart rate variability (HRV) indices in adults. However, to our knowledge there are no studies that have monitored ballet training loads concomitantly with possible changes in cardiac autonomic control and, therefore, it is still not clear whether ballet training causes positive or negative HRV responses in female adolescents.

Furthermore, the adaptations to physical training during puberty are also modulated by a fine balance between anabolic and catabolic hormones (Roemmich et al., 2001; Eliakim et al., 2013). Due to the high physical and physiological demands of ballet in combination with diet restriction and weight control (Zoletic et al., 2009), ballerinas are considered risk groups for the development of disturbances in the hypothalamic-pituitary-gonadal axis (Roemmich et al., 2001). In this respect, hematological and hormonal changes are regarded as either harmful or beneficial states, temporary or otherwise, that could lead to health problems, particularly during puberty, which is considered a distinctive period of physical growth and development (Malina et al., 2000). Specifically in females, the androgens have a direct and crucial effect on bone mass, muscle growth and erythrocyte production, and the load of physical exercise may play an important role in these interactions (Enea et al., 2011). Thus, the purpose of this study was to examine the possible alterations in autonomic modulation, body composition, the hormonal profile and hematological indices after 17-weeks of ballet training planned in preparation for a high-level international competition.

## Material and Methods

### Experimental Approach to the Problem

This longitudinal descriptive study followed 17 weeks of ballet training planned as preparation for a high-level international competition. Pre- and post-training period data were obtained in accordance with the following steps: firstly, blood samples were collected for hematological and hormonal markers; secondly, dancers rested for a 10-min period in order to obtain resting heart rate data; finally, anthropometric and body composition measurements were performed. During the training period, with the aim of following the physical intensity of each training session, the dancers’ session rating of perceived exertion (session-RPE) was obtained 30 min after the completion of every training session. The baseline data acquisition was performed on Monday morning prior to the start of the first ballet training session after a 20-day holiday period. The post-training data collection was performed 17 weeks later, 48 h after the final training session.

### Subjects

Twenty-four female pre-professional ballet dancers (12–15 years of age) with 5 years of classical ballet experience volunteered to take part in the study. The dancers had trained for at least 10 hours per week throughout the previous year. All participants and their parents gave signed consent and the procedures were in accordance with the Research Ethics Committee of the State University of North Paraná. Medical screening was performed to verify whether all the adolescents were healthy, at most one year postmenarcheal and had a good nutritional status, which was assessed through a recorded inquiry. None of the subjects demonstrated menstrual abnormalities (amenorrhea or oligomenorrhea), eating disorders (i.e., anorexia and/or bulimia nervosa) or pharmacological intake during the study period. Likewise, they were free of cardiovascular, autonomic and metabolic dysfunction, and were engaged only in dance activities. All adolescents reported that they neither smoked nor ingested alcoholic beverages during the study period. Table 1 presents the characteristics of the participants.

### Procedures

#### Body Composition

Body mass and height were measured (pre and post) using a scale and a stadiometer to the nearest 0.02 kg and 0.1 cm, respectively. Skinfold thicknesses were measured to the nearest 0.2 mm using a caliper on the right side of the body. The subscapular and tricipital skinfolds were measured three times and the average of the three values was recorded. The Slaughter’s equation was applied to calculate lean body mass (kg) and relative body fat (%). This equation was chosen because it had been specifically proposed for use in children and adolescents (Slaughter et al., 1988).

#### Training

Throughout the 17 training weeks the sessions were structured into three parts: firstly, ballerinas performed barre exercises composed of one leg, arms and rotation around the body axis, all performed with one hand touching the barre throughout the five feet positions, with increasing technical difficulty, including *pliés, grands battements, battements jetés, frappés and foundus*. Secondly, they performed center floor exercises (without touching the barre) such as intense *temps levés* or *sautés* (jumping at “allegro”) followed by *grand adage on full pointe*. In this part, the ballerinas performed *grand adage* using both sides (right and left) and pirouette sequences (2, 3 and 4 tours) on full pointe. The final part lasted about 2 hours and was composed of choreography practice and rehearsals. During the rehearsals all the ballerinas performed choreographies in groups and twelve of them rehearsed ballet repertoires (individual). The total training time was at least 10 hours per week.

#### Internal training load

The internal training load was obtained using the session-RPE method. Approximately 30-min following the completion of each ballet class, the adolescents were required to rate the perceived intensity of the whole class on a category ratio (CR10) Borg scale (1998). From the session-RPE method, monotony and strain were also calculated (Foster et al., 1998). Monotony was calculated by dividing the mean daily load over each week by its standard deviation. Strain was calculated by multiplying monotony by the accumulated weekly training load (Foster et al., 1998). Monotony reflects the variability of training loads while strain represents the general stress caused by the loading and low variability of the practice sessions. All the ballerinas had been previously familiarized with the use of the RPE scale and the results were recorded individually in a notebook without interference of colleagues or the coach.

#### Biochemical markers

Pre- and post-training blood samples were collected in the morning (between 7 a.m. and 9 a.m.), after 8 hours of overnight fasting, using a vacuum venous puncture. The ballerinas did not perform any physical exercise for 48 hours prior to blood collection either in the pre or post-training moments. Laboratory analyses of biochemical variables (urea, creatinine, uric acid, total protein, albumin and creatine kinase) were performed using the dry-chemistry method (Fusion. Johnson & Johnson do Brasil, São Paulo, Brazil) and C-reactive protein (CRP) was measured by the highly-sensitive chemiluminescent method (Siemens Diagnostics, Munich, Germany). Samples of blood counts were analyzed by the electrical impedance method using the Pentra-120 brand Horiba ABX device. Hormones (testosterone, cortisol and thyroxine) and ferritin were analyzed using the chemiluminescent method (Architect-Abbott, Illinois, USA).

#### Cardiac autonomic modulation

For this purpose, the duration of the RR interval recordings (RRinterval) was obtained from each ballerina using a portable heart rate monitor (Polar RS800, Kempele, Finland). The recordings were downloaded via commercial software and exported for later analysis of time and frequency domain measures of HRV. The time domain indices examined were: the mean RR interval, the root-mean-square difference of successive normal RR intervals (RMSSD), which reflects vagal modulation, and the standard deviation of all normal RR intervals (SDNN), which comprises both sympathetic and vagal cardiac modulations (Task Force, 1996). During the 10-min resting period, the ballerinas laid in the supine position, breathing spontaneously. The final 5-min stationary period was analyzed for HRV with all RR intervals visually inspected and ectopic beats (<3%) manually removed and replaced by the interpolation of adjacent RR intervals.

### Statistical Analyses

Data distribution was verified using the Shapiro-Wilk’s test. Where appropriate, either the Student’s t test or Wilcoxon (Z) test were applied to detect differences in the considered variables. Repeated measures ANOVA with Bonferroni correction was used to compare the session-RPE (in arbitrary units, a.u.) for every training week (mean session-RPE, sum of session-RPE, monotony and strain). The Pearson product-moment correlation was used to correlate loads measured by the RPE-method with the biochemical indices and autonomic modulation. The significance level was set at 5%.

## Results

Table 1 displays the anthropometric and body composition variables pre- and post-ballet training. None of the initial 25 adolescents was excluded from the final analysis. There were significant increases in body mass (p = 0.02) and height (p < 0.01) after the 17 training weeks. In addition, there was a decrease in relative body fat and an increase in lean body mass (p < 0.01).

**Figure 1:**
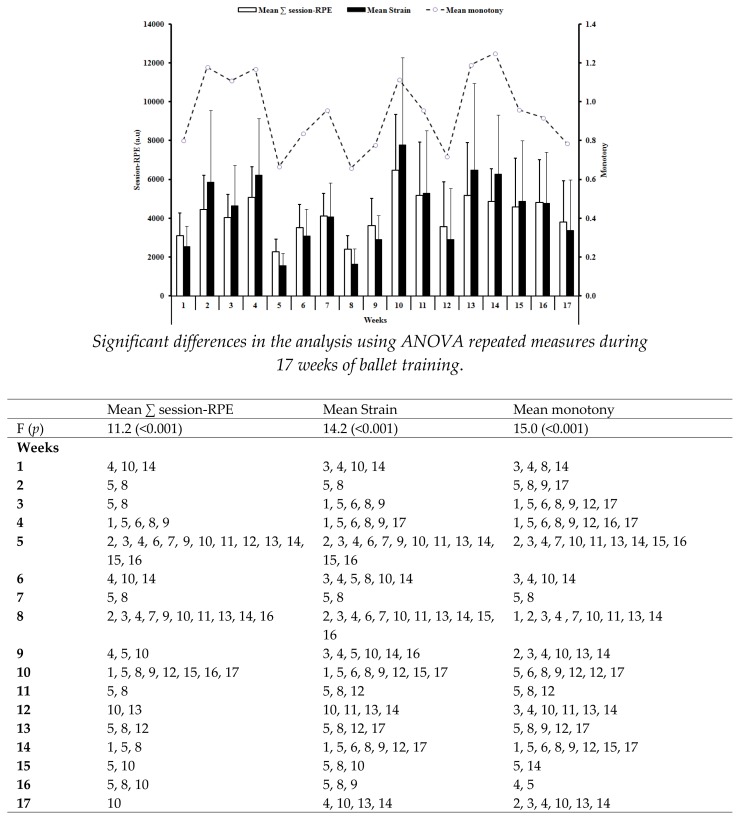
Weekly periodization determined using mean weekly RPE-based training load (session-RPE) during the 17 wks of ballet training (n=24); a.u.=arbitrary unit.

During the training weeks, the ballerinas trained for mean duration of 15.3 ± 3.3 hours per week. The mean weekly training load over the 17 weeks was 596 ± 153 a.u. and the mean total weekly training load was 4.175 ± 1.071 a.u.. The means of strain and monotony obtained were 4.365 ± 1.810 and 0.9 ± 0.2, respectively. Throughout the 17 weeks, there was a large variation in daily session-RPE with differences observed in all measurements: mean ∑ session-RPE (F=11.2; p < 0.01); mean strain (F= 14.2; p < 0.01) and mean monotony (F=15.0; p < 0.01) (Figure 1).

Biochemical markers showed significant changes between the moments. Table 2 shows that there were significant increases in total protein and urea (p < 0.01) while there was a decrease in uric acid (p < 0.01) after the training period. C-reactive protein also increased after the training period (p < 0.01). Regarding the hematological profile, there was a slight decline in red blood cell count concomitantly with an increase in hemoglobin concentration (p < 0.05), while ferritin presented a decrease after the training period (p < 0.05). There were no significant changes between moments for white blood cell count. Concerning the hormonal responses; testosterone, the testosterone/cortisol ratio and thyroxine demonstrated significant increases (p < 0.01).

Table 3 shows the HRV values pre- and post-training. There were significant increases in the RR intervals and RMSSD after the 17 weeks of ballet training (p < 0.01). Additionally, there were reductions in LF (% change −6.7; p < 0.05) and there was an increase in the HF parasympathetic index (% change 22.6; p < 0.05) after the training period. These alterations led to a significant decline in the LF/HF (% change −5.8; p < 0.05).

Finally, no differences were found between biological markers and the training loads measured by the RPE-method.

## Discussion

The main findings of this study were that after 17 weeks of ballet training a decrease in relative body fat and an increase in lean body mass of ballerinas concomitant with an increase and decrease in testosterone and cortisol, respectively, were observed. Likewise, after the 17 weeks of training, improvements in HRV vagal-related indices such as RMSSD, HF and LF/HF were found. These results suggest that training loads did not negatively affect physical growth, body composition or the endocrine and autonomic systems. Furthermore, no significant relationships were observed between the positive biological adaptations and training loads measured by the RPE-method.

Classical and contemporary ballet are widely acknowledged as predominantly intermittent exercises (Twitchett et al., 2011; Rodrigues-Krause et al., 2014) with performance imposing high demands on the aerobic and anaerobic systems (Bria et al., 2011; Twitchett et al., 2011).

For instance, Guidetti et al. (2008 and 2009) showed VO_2_ responses which were often above the individual anaerobic threshold, coupled with a high mean heart rate and blood lactate concentration, during a typical ballet lesson of females between 13 and 16 years old. Additionally, relative to body mass, VO_2_ and blood lactate concentrations during ballet classes are different in dancers of a low technical level compared to those observed in their high-level counterparts. This is consistent with previous findings in inexperienced and experienced aerobic dancers (Laukkanen et al., 2001). Recent studies have suggested that the physiological responses during 45-min of ballet class and a rehearsal for the Paquita repertoire demonstrated that the rehearsal was more demanding than the classes (i.e., higher VO_2_, heart rate and blood lactate responses) (Rodrigues-Krause et al., 2014). In the present study, the pattern of the weekly loads imposed on the organism of dancers was measured during all the ballet training sessions in preparation for an important competition (Figure 1). Throughout the training period there were several changes in weekly training loads reported by the dancers. For instance, from the fifth to ninth week there was a decrease in loads followed by an increase from the tenth until the last week of training. Additionally, a large variation in loads was noted throughout the 17 weeks of training with an average monotony of 0.94 and strain of 4365 (a.u.). These values of monotony were lower (1.11) while the strain was similar (average 4262 a.u.) to that experienced by young female elite athletes involved in different sports (team sports, racket sports and individual modalities) (Malisoux et al., 2013). Foster et al. (1998) suggested a relationship between the training loads, strain, and monotony of practices and the likelihood of overreaching/overtraining. The results of the present study demonstrated that despite an elevated training volume (~15 hours per week) and an average rating of perceived effort of 5.8 (intense level), the ballerinas presented positive adaptations to training, without injuries, complaints or any evidence of either non-functional overreaching or overtraining. It is also important to note the great intra-variability between the ballerinas (e.g. high standard deviation) during the observed weeks. This great variability indicates differences in the internal training load perceived by the dancers which may reflect individual differences in technical and artistic skills; dancers with higher skills achieve perfection by undertaking fewer exercises compared to their peers and, therefore, report lower values of perceived exertion during the sessions. The variable load distribution between classes and rehearsals may have reflected positively on the autonomic nervous system of the ballerinas. To access the influence of training on cardiac autonomic responses we used HRV analysis (Task Force, 1996). Previous studies have found that HRV provides important indices for the identification of training stress (Aubert et al., 2003) and the course of recovery for optimal training adaptations of adult athletes (Atlaouli et al., 2007). Heart rate variability could also be used as a tool for monitoring training responses (Eliakim et al., 2013). However, few studies have reported the responses of HRV indices, after training, in pediatric populations (Silva et al., 2014). Similar to the present study, Triposkiadis et al. (2002) investigated the effects of intensive training on prepubertal swimmers and reported increased HRV after the training period. Additionally, Sartor et al. (2013) also showed improved HRV after 10 weeks of physical training in elite 16 year old male gymnasts. The present results strengthen the positive influence of training on resting cardiac autonomic modulation of adolescents. Improvement in HRV has been related to reduced anxiety and improved artistry in competitive ballroom dancers (Gruzelier et al., 2014).

Regarding the positive adaptations of body composition and anthropometry, the findings of the present study suggest that training may have been a determinant of high plasma concentration of testosterone and thyroxine. In adolescents, increased levels of circulating testosterone and a higher testosterone/cortisol ratio are associated with performance improvements (Moreira et al., 2013). Significant increases in testosterone (36.5 to 43.7 ng/dL; *p* < 0.01) and a non-significant decrease in cortisol were demonstrated in the current study. Additionally, the testosterone/cortisol ratio was higher at the post-compared to the pre-training moment (3.25 to 3.99; *p* = 0.02). These changes suggest that after the training period female adolescent ballet dancers reached an anabolic state favorable to increases in muscle mass and physical performance, in a similar fashion to a previous study involving adolescent female gymnasts (Jahreis et al., 1991). Furthermore, the alterations in blood creatine kinase concentration in this study were not statistically significant, allowing us to conclude that after the training period the ballerinas did not present signs of muscle damage. Likewise, the results demonstrated a significant decrease in CRP concentration (0.52 to 0.07; *p*<0.01) which suggests that a low degree of inflammation was present after the ballet training period. This may have occurred due to the protective effect of exercise (Marklund et al., 2013) throughout the 17 weeks of training, resulting in decreases in muscle damage and inflammation which is a consequence of chronic stress exposure in ballroom dancers (Berndt et al., 2012).

According to the hormonal and biochemical profile, there was a decrease in relative body fat (19.7 to 17.40%) concomitant with an increase in lean body mass (34.9 to 37.0 kg), demonstrating a positive effect of ballet training on body composition. Results of relative body fat in the present study were lower (17.4 ± 4.6%) than those presented by Eliakim et al. (2000) in adolescent ballerinas (22.5 ± 0.01%), who had also trained ~15 hours per week. Additionally, our data demonstrated similar values of lean body mass (kg) and relative body fat (%) compared to adult ballet dancers (26.6 ± 4.4 years) from Japan (Kuno et al., 1996). Increases in the ballerinas’ body mass and height after the training period were also noted (Table 1). This morphological alteration was expected due to the intense longitudinal skeletal growth that occurs during this period (Malina et al., 2011), and therefore took place independent to the ballet training as shown by Steinberg et al. (2006). This latter study involved 1,482 female teenagers in a cross-sectional study and no alterations in growth and biological maturation between ballet dancers and non-dancers of 8 to 16 years old were found.

In the present study an increase in urea (25.5 to 29.3 mg/dL; *p* < 0.01) and a decrease in uric acid concentrations (4.2 to 3.7 mg/dL; *p* < 0.01) was also observed after the training period. Similar findings have been reported in elite adult soccer players during the competitive season (Marklund et al., 2013). Elevations in uric acid levels were noticed after intense aerobic (Aguiló et al., 2005) and anaerobic (Groussard et al., 2003) exercises and reflected an increased purine metabolism or decreased renal uric acid clearance (Groussard et al., 2003). The lower uric acid levels post-training might reflect the lower training volume undertaken by the ballerinas in the final three weeks of training (Figure 1). Regarding urea concentration, the most common reason for this rise in elite athletes is the training volume associated to some degree with glyconeogenesis and protein degradation (Urhausen et al., 1992). Nevertheless, due to the increases in total protein and albumin, and to the maintenance of creatinine concentration, which ranged within normal values pre- and post-training (Table 2), catabolic events might not be the explanation for the increased levels of urea in the present study. The results of the present study are in discrepancy with those of Filaire et al. (2003), who showed that after 16 weeks of training in seven international level female artistic gymnasts (about 14.5 years) there were significant increases in uric acid, creatinine and cortisol, highlighting the catabolic impact of training. Regarding the hematological parameters, training is known to cause plasma volume expansion and a consequent decrease in red blood cell count and the fraction of the hemoglobin to plasma volume relationship (Neumayr et al., 2005). Intense training periods also appear to contribute to a reduction in circulating levels of ferritin (Koehler et al., 2012). Female athletes particularly are prone to nonanemic iron deficiency. In the present study, a trend in the development of sports pseudoanemia and iron deficiency was observed. Similar results were found in young gymnasts (Constantini et al., 2000). Thus, special attention should be paid to dietary requirements in young female athletes, including ballerinas, in order to avoid the risk of iron deficiency (Koehler et al., 2012).

We recognize that the absence of a control group may be considered a limitation of this study. However, it is not possible to exclude ballerinas from the training routine in order to compose a control group. Likewise, it is very difficult to find female counterparts with similar characteristics to the ballerinas. Nevertheless, the key features of the results reported here may provide clues when planning training.

In conclusion, seventeen weeks of ballet training, including ballet classes and rehearsals, resulted in improvements in body composition and cardiac autonomic modulation in young dancers. Blood parameters contributed to explaining the positive impact of a training routine that preceded a ballet competition in female adolescents. Additionally, the adolescent ballerinas demonstrated normal physical growth and strong signs of anabolism during the monitored period.

## Practical Applications

The current study is the first to describe the dancers’ perceived load throughout 17 weeks of training for an important competition. Coaches can benefit from this information by creating early interventions with a reduced risk of an excessive or insufficient stimulus and focusing on the individualization of physical loads according to the ballerinas’ perceived exertion. The high inter individual variability in the loads might reflect the differences in technical and artistic skills, reflecting a higher exigency for the dancers who present lower artistic competence.

## Figures and Tables

**Table 1:** Indicators of physical characteristics and body composition assessed before and after (Post) training (n=24) (mean±SD)

	Pre Training	Post Training	*p*
Chronological age (years)	13.2 ± 1.4	13.7 ± 1.5	0.06
Body mass (kg)	43.9 ± 6.3	45.2 ± 6.5	0.02*
Body height (m)	1.55 ± 0.04	1.58 ± 0.04	<0.01*
BMI (kg/m^2^)	18.0 ± 2.3	17.1 ±2.3	0.70
Relative body fat (%)	19.7 ± 4.5	17.4 ± 4.6	<0.01*
Lean body mass (kg)	34.9 ± 3.92	37.0 ± 4.3	<0.01*

T test - dependent samples (*P<0.05)

**Table 2:** Median values (M) and interquartile range (Q1 – Q3) of biochemical variables. Blood count and hormones assessed before and after (Post) training.

	Pre (January)	Post (July)	*p*
*Biochemical variables*			
Urea (mg/dL)	25.5 (19 – 31)	29.3 (23 – 34)	<0.01*
Creatinine (mg/dL)	0.66 (0.6 – 0.7)	0.67 (0.6 – 0.7)	0.72
Uric Acid (mg/dL)	4.2 (3.6 – 4.8)	3.7 (3.3 – 4.2)	<0.01*
Total Protein (g/dL)	7.6 (7.4 – 7.9)	8.1 (7.8 – 8.4)	<0.01*
Albumin (g/dL)	4.5 (4.4 – 4.7)	4.7 (4.6 – 4.8)	0.02*
CK (U/L)	87.1 (64 – 97)	81.5 (62 – 88)	0.40
CRP (mg/dL)	0.52 (0.50 – 0.7)	0.07 (0.0 – 0.10)	<0.01*
Ferritin (ng/mL)	43.2 (28.8 – 55.2)	36.9 (27.7 – 43.3)	0.02*

*Haemogram*			
Red blood cell ^(million/mm3)^	4.7 (4.5 – 5.0)	4.6 (4.4 – 4.8)	<0.01*
Hematocrit (%)	40.5 (39.3 – 42.0)	40.7 (39.3 – 42.0)	0.80
Hemoglobin (g/dL)	13.3 (12.8 – 13.9)	13.8 (13.3 – 14.3)	<0.01*
Plaquets ^(X103/mm3)^	255 (225 – 290)	257 (231 – 284)	0.67
White blood cell^(x103/mm3)^	6.6 (5.9 – 7.3)	6.9 (6.0 – 7.6)	0.63
Neutrophils (%)	48.1 (44.9 – 56.4)	50.3 (43.3 – 57.5)	0.13
Lymphocytes (%)	42.0 (33.6 – 47.2)	39.0 (33.7 – 45.8)	0.09
Monocytes (%)	6.5 (5.6 – 7.2)	6.8 (5.8 – 7.5)	0.20

*Hormones*			
Cortisol (μg/dL)	12.5 (8.3 – 16.2)	12.2 (8.0 – 15.0)	0.94
Estradiol (μg/dL)	64.0 (30 – 91)	69.2 (29 – 61.5)	0.85
Testosterone (ng/dL)	36.5 (20 – 51)	43.7 (28 – 50)	<0.01*
T/C ratio	3.25 (1.6 – 3.8)	3.99 (2.2 – 5.2)	0.02*
TSH (ng/dL)	3.94 (1.7 – 5.1)	2.6 (1.3 – 3.38)	0.19
T4 (ng/dL)	1.0 (0.97 – 1.12)	1.23 (1.11 – 1.31)	<0.01*

Wilcoxon Test - dependent samples (*p < 0.05)

**Table 3:** Indicators of heart rate variability assessed before and after (Post) training (n=24) in median, first and third quartile intervals.

	Pre (January)	Post (July)	*p*
HR mean (bpm)	84.8 (76.6 – 91.7)	76.8 (69.6 – 82.7)	<0.01*
RR mean	712.4 (659.4 – 795.1)	786.0 (727.6 – 865.5)	<0.01*
SDNN	39.4 (31.9 – 60.0)	46.0 (34.1 – 61.4)	0.12
RMSSD	36.8 (27.2 – 72.8)	59.7 (37.1 – 74.6)	<0.01*
LF (ln·ms^2^)	6.5 (5.9 – 7.0)	6.6 (5.9 – 6.9)	0.75
LFnu	45.4 (32.9 – 63.2)	40.4 (33.7 – 53.4)	<0.05*
HF (ln·ms^2^)	6.5 (6.0 – 7.3)	7.1 (6.1 – 7.3)	<0.05
HFnu	54.6 (36.8 – 67.2)	59.6 (46.6 – 66.3)	<0.05*
LF/HF	0.9 (0.5 – 1.7)	0.7 (0.5 – 1.1)	<0.05*

Wilcoxon Test - dependent samples (*p < 0.05)
